# Upregulation of miR-20a-5p as the Potential MicroRNA Marker in Red Blood Cell Storage Lesion

**DOI:** 10.1155/2023/5598590

**Published:** 2023-10-04

**Authors:** Yanisa Rattanapan, Sodsai Narkpetch, Takol Chareonsirisuthigul

**Affiliations:** ^1^Medical Technology, School of Allied Health Sciences, Walailak University, Tha Sala, Nakhon Si Thammarat, Thailand; ^2^Hematology and Transfusion Science Research Center, Walailak University, Nakhon Si Thammarat 80160, Thailand; ^3^Blood Bank, Maharaj Nakhon Si Thammarat Hospital, Nakhon Si Thammarat 80000, Thailand; ^4^Department of Pathology, Faculty of Medicine Ramathibodi Hospital, Mahidol University, Bangkok 10400, Thailand

## Abstract

**Background:**

Packed red blood cells (PRBCs) can be preserved for 42 days, and stored PRBCs have slow, dangerous changes over time during storage. miRNA is approximately 22 nucleotides long, a small single-stranded noncoding RNA molecule. miRNA guides by pairing bases with their downstream target mRNA to regulate negative expression. They are essential in many life processes, including cell differentiation, proliferation, and apoptosis. Therefore, miRNA alterations may represent possible biomarkers of PRBC storage lesions. This study is aimed at validating the miR-20a-5p in PRBC storage. *Study Design and Methods*. A total of 20 PRBC samples were divided into day 1 and day 20 storage groups. Total miRNA was extracted and quantified by probe-based RT-qPCR assays to explore the potential role of miRNAs in PRBC storage lesions.

**Results:**

Upregulated miR-20a-5p in PRBC storage on day 20 compared to day 1. MiR-20a-5p promoted cell survival, which may affect the downstream regulation and decrease PRBC viability in prolonged storage.

**Conclusion:**

On this basis, this detection may help to assess the quality of stored PRBCs.

## 1. Introduction

Packed red blood cells (PRBCs) are red blood cells separated for blood transfusion [1]. PRBCs are often used in anemia or when the hemoglobin is below 7-8 g/dL [1–3]. Whole blood is often collected from donated blood and spun in a centrifuge. Red blood cells (RBCs) become denser and fall to the bottom, and most liquid blood plasma remains on top. Plasma is separated, and the RBCs are kept with the lowest amount of fluid. The additive solution of citrate, dextrose, and adenine is generally mixed with cells to keep them alive during storage. With a general additive solution, PRBCs are stored in blood bank refrigerators for up to 42 days at 1-6°C [4]. In some patients, using much fresher PRBCs is essential. Neonates should receive blood collected in less than seven days for optimal cell function. Prolonged storage of transfused PRBCs has been associated with hemolysis in healthy adults and inflammation in animal models [5]. Rupture of prolonged storage PRBCs can release miRNA into the plasma, a severe complication that can occur after a blood transfusion [6, 7]. However, PRBC storage lesions and their implications for transfusion efficacy are complex and controversial.

MicroRNA (miRNA or miR) regulates the expression of genes involved in differentiation and apoptosis through mRNA degradation or translation inhibition. miRNAs are also implicated in various diseases and can be used as biomarkers. Various miRNAs exist in mature erythrocytes, but their functions are unknown. miRNA expression in erythrocytes differed from that in reticulocytes or white blood cells. Nuclear-forming cells have significantly higher miRNA content, but the hematopoietic cellular contribution to miRNA content of blood on a volume basis is highest in erythrocytes. Therefore, miRNAs will likely play a crucial role in posttranscriptional regulation in erythroid cells [8, 9].

miR-20a-5p was ranked among the top three of the top 22 upregulated miRNAs, with the greatest *p* values of the changes in the miRNA expression profile during blood storage [10]. mir-20a-5p is a potentially crucial key regulator sensitive to the storage condition of human platelets used for transfusion. Interestingly, some miRNAs are upregulated in red blood cells (anucleate cells) during 4°C storage [11]. It remains unclear whether the observed differential miRNA expression is universally due to the degradation of miRNAs because of storage. Therefore, differential miRNA expression is essential because anucleate cells such as platelets and red blood cells should not actively synthesize miRNAs, indicating that degradation may significantly contribute to increases in miRNAs. Accordingly, it is possible that differential miRNA expression can be seen in other cell types under storage at 4°C and that this is not necessarily specific to platelets [12].

Dysregulated miRNAs represent potential biomarkers to identify storage lesions, and their detection helps evaluate the quality of stored PRBCs and prevent acute hemolytic transfusion reaction (AHTR). However, neither of these miR-20a-5p of target mRNAs have been reported. Therefore, this study is aimed at validating the miR-20a-5p in PRBC storage in a different cohort. The relative expression of this miRNA was analyzed, and the possible mechanisms were discussed to provide further insights into PRBC apoptosis during storage.

## 2. Materials and Methods

### 2.1. Sample Collection

A total of 40 packed red blood cell (PRBC) samples were collected from healthy voluntary blood donors who passed the initial screening from the blood donation application form, National Blood Centre, Thai Red Cross Society. 350 to 450 mL of blood was collected into 63 mL citrate phosphate dextrose adenine (CPDA-1). Blood samples that did not meet the required volume, hemolysis, icterus, and lipemia (HIL) were excluded. In the process of PRBC preparation, the whole blood from a double blood bag was subjected to the removal of plasma by centrifugation at 1700 rpm, 22°C for 7 minutes, which is often followed by the removal of white cells by filtration. Each PRBC sample was stored at 4°C and divided into two conditions: fresh PRBCs on day 1 and stored PRBCs on day 20. PRBCs were transferred into 6 mL K3-EDTA for miRNA extraction at the appointed date.

### 2.2. RNA Extraction

Isolation RNA from 350 *μ*L PRBCs using the RNeasy® Mini Kit (Cat. Nos. 74104 and 74106) (QIAGEN, Hilden, Germany) according to the manufacturer's instructions. Quantification and the purity of RNA were evaluated using NanoDrop™ One/OneC Microvolume UV-Vis Spectrophotometer (Thermo Fisher Scientific, Waltham, Massachusetts, United States).

### 2.3. cDNA Synthesis of Mature miRNAs

Each template RNA sample was diluted to 5 ng/*μ*L using nuclease-free water. Reverse transcription (RT) reaction master mix was performed using the miRCURY LNA RT Kit protocol (QIAGEN, Düsseldorf, Germany). RT reaction temperature cycling steps were RT step, inactivation of reaction, and storage: 42°C for 60 min, 95°C for 5 min, and 4°C forever, respectively. The reactions were reacted using Veriti™ 96-Well Fast Thermal Cycler (Thermo Fisher Scientific, Waltham, Massachusetts, United States). Storage undiluted cDNA at 2-8°C for up to 4 days or at -30 to -15°C for up to 5 weeks until use.

### 2.4. miRNA RT-qPCR

miRCURY LNA SYBR Green PCR was duplicated in 96-well plates. cDNA from each sample was diluted at 1 : 60, and the reaction mix for miRCURY LNA miRNA PCR Assays was performed according to the manufacturing protocol (QIAGEN, Hilden, Germany). The PCR cycling conditions were PCR initial heat activation, 2-step cycling with 40 cycles (denaturation and combined annealing/extension), 95°C for 2 min, 95°C for 10 s, and 56°C for 60 s, respectively. The melting curve analysis was 60-95°C. The reactions were reacted using StepOnePlus™ Real-Time PCR System (Thermo Fisher Scientific, Waltham, Massachusetts, United States). Primers were used from the miRCURY LNATM miRNA PCR Assay, hsa-miR-20a-5p (Cat. No. YP00204292), and normalized to the stably expressed reference U6 snRNA (Cat. No. YP00203907) (QIAGEN, Hilden, Germany).

### 2.5. miRNA Target Gene Prediction

Target mRNAs were obtained by the miRDB web-based database (http://mirdb.org/). Enter the miRNA that needs to know its target mRNA into the microRNA name field and click Go. The target mRNA search query for that miRNA was displayed. Data were manually selected from miRNAs that target cellular necrosis, O_2_ delivery, inflammation, and apoptosis pathways.

### 2.6. Statistical Analysis

CT mean was determined from the duplicate experiments. The comparative CT (2^–△△CT^) method analyzes comparative gene expression data. U6 snRNA was used as an internal control for miRNA qPCR assay. Data were summarized by giving their average and standard deviation (SD), and student's *t*-test was used to compare the means of the two samples of related data. Statistical analysis was performed using SPSS version 20.0 (IBM, Armonk, NY, USA). Statistical significance was defined at *p* < 0.05.

## 3. Results

A total of 20 donated blood donors were male and female, 17 (85%) and 3 (15%), respectively. The median age was 38 (22-57) years. The highest number of blood groups was O 10 (50%), followed by A 5 (25%), AB 3 (15%), and B 2 (10%), respectively. Twenty donated blood was separated two times for miRNA extraction: day 1 as the fresh blood control and day 20 as the average blood unit at the time of transfusion (Table 1).

**Figure 1:**
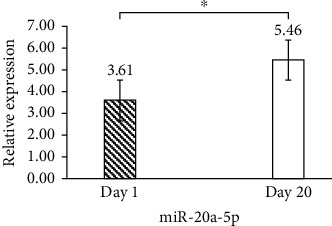
Increased expression of miR-20a-5p in 20-day PRBCs stored compared with one-day (control) storage using RT-qPCR assay.

The miR-20a-5p expression on day 1 (control) and day 20 stored packed red blood cells (PRBCs) was analyzed using RT-qPCR assays. It increased expression in PRBCs stored for 20 days (5.46 ± 11.05) compared with one-day storage (3.61 ± 6.43) with a *p* value = 0.02 (Figure 1).

**Figure 2:**
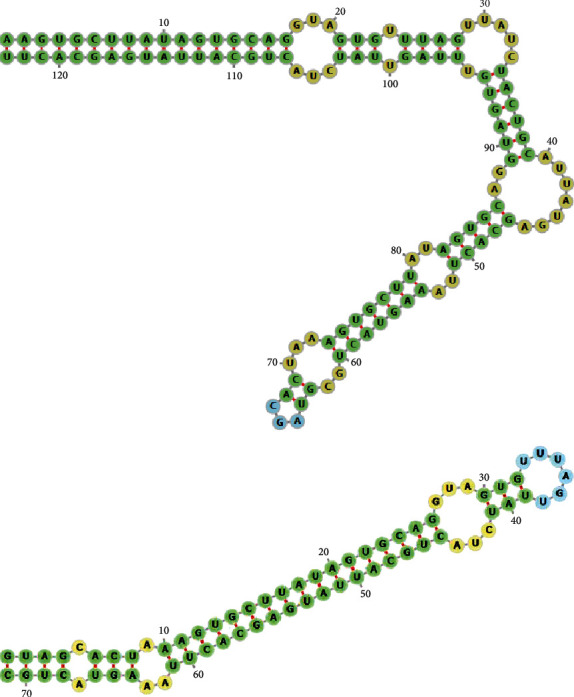
Predicted microRNA hairpin structures of miR-20a-5p.

Predicted precursor-miR-20a-5p (pre-miR-20a-5p) contains 123 (top) and 71 (bottom) nucleotides, respectively, and folds into a hairpin structure by using miRNAFold (https://evryrna.ibisc.univ-evry.fr/miRNAFold) (Figure 2). Mature miR-20a-5p sequences were 23 nucleotides long as UAAAGUGCUUAUAGUGCAGGUAG at nucleotide position 70-92 (top) and 8-30 (bottom), respectively.

MiR-20a-5p predicted their biological targets and functional annotations using TargetScan (https://www.targetscan.org/vert_80/) and miRDB (https://mirdb.org/). TargetScan showed 112 target genes, while miRDB showed 1381 target genes. All target genes were sorted into duplicates resulting in the remaining 79 target genes. Seventy-nine target genes were selected, leaving only 22 annotated genes based on structural, biochemical, and immunological changes using HGNC (https://www.genenames.org/) (Table 2).

**Figure 3:**
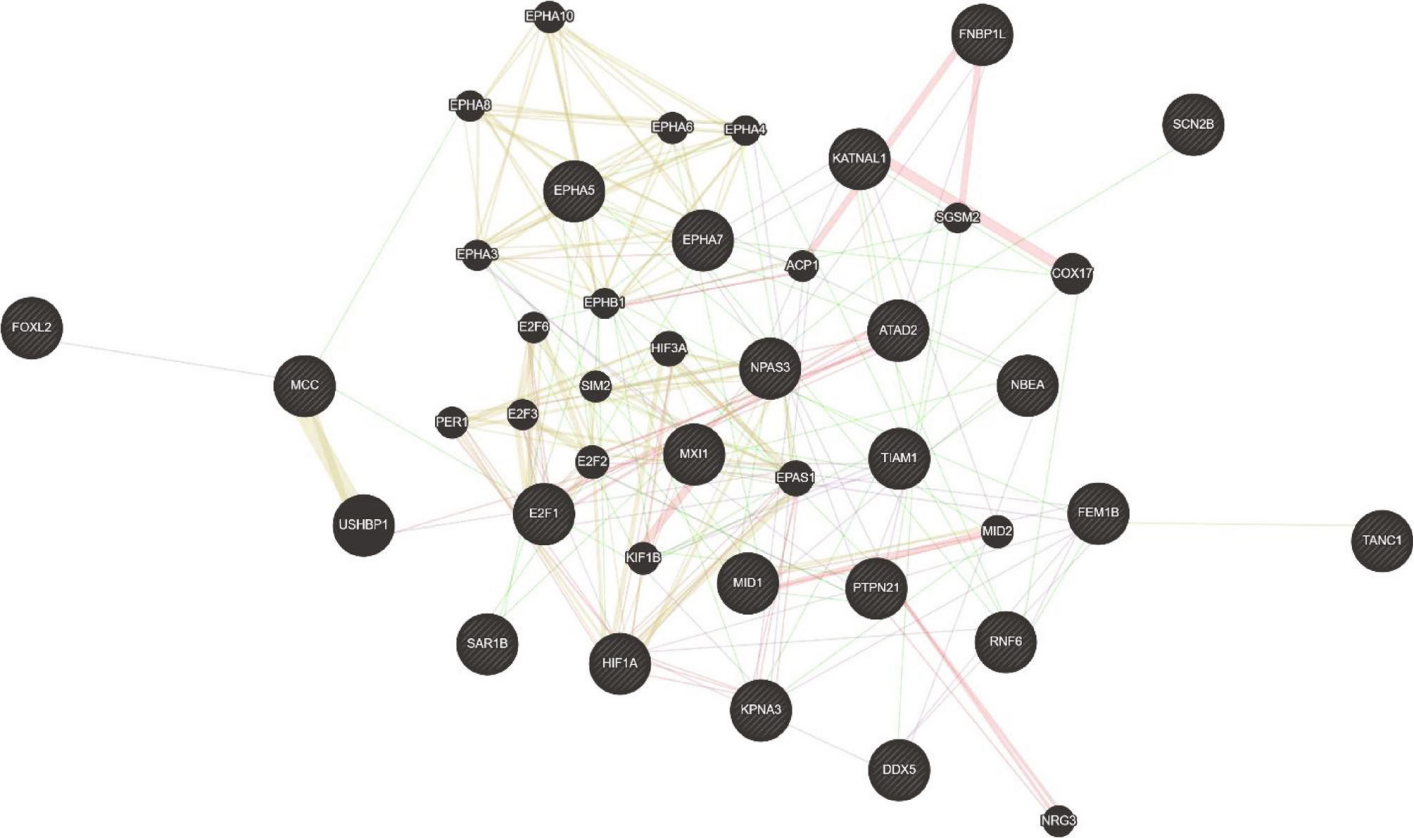
Coexpression network of potential target genes of miR-20a-5p.

Twenty-two target genes of miR-20a-5p in PRBC storage lesion predicted their association network based on the automatically selected weighting method using GeneMANIA (https://genemania.org/) (Figure 3).

## 4. Discussion

As the stored blood ages, the energy source within PRBCs is depleted, reducing the integrity of the membrane structure. Therefore, stored red blood cells become deformed and more fragile with age, leading to the release of cell-free hemoglobin and the formation of microparticles. Since the biochemical alterations of the PRBC unit shift over time, the transfusion of older blood products may contribute to hemolysis and the release of hemoglobin, resulting in increased nitric oxide (NO) intake. NO contributes to many activities linked to the storage lesions, including blood flow, inflammation, and thrombosis [13]. These abnormalities may cause cardiac complications, including hyperkalemia and cardiac arrest [14].

miRNA is approximately 22 nucleotides long, a small single-stranded noncoding RNA molecule. miRNA guides by pairing bases with their target mRNA to regulate negative expression. They are essential in many life processes, including cell differentiation, proliferation, and apoptosis. Therefore, miRNA alterations may represent possible biomarkers of PRBC storage lesions [11].

This study recruited miR-20a-5p with significantly different expressions in PRBCs stored between 1 and 20 days. This miRNA was ranked among the top three of the top 22 upregulated miRNAs with the greatest *p* values of the changes in the miRNA expression profile during blood storage [10]. However, neither of these miR-20a-5p of target mRNAs have been reported. Therefore, this study validated miR-20a-5p and explored the potential role of miRNAs in PRBC storage lesions.

miR-20a is a miR-17/92 cluster member located in the 13q31.1 region, mainly associated with inflammation. Overexpression of miR20a could reduce inflammasome NLRP3 activity by mediating targeting thioredoxin-interacting protein (TXNIP). miR-20a also regulates signal-regulatory protein *α* (SIRP*α*), resulting in macrophage infiltration, phagocytosis, and proinflammatory cytokine secretion [15]. Additionally, miR-20a-5p may play an important role in cell proliferation, invasion, and metastasis in various cancers [16]. In head and neck squamous cell carcinoma (HNSCC), miR-20a-5p functioned as an oncogene by downregulating TNFRSF21 and upregulating CCR7 expression [17]. In bladder cancer, miR-20a-5p promoted growth and metastasis by inhibiting the expression of the tumor suppressor gene NR4A3 [18]. This study showed the upregulated miR-20a-5p with their potential target on structural, biochemical, and immunological changes in PRBC storage lesions.

PRBC storage on day 20 compared to day 1 found that the miR-20a-5p was significantly upregulated. miR-20a-5p promoted cell survival, which may affect the downstream regulation and decrease PRBC viability in prolonged storage. Based on the results, 22 genes were predicted as candidate target genes of miR-20a-5p. These target gene types are protein-coding that might be involved in DNA replication, cell cycle regulation, RNA metabolism, cell growth, proliferation, differentiation, longevity, and ubiquitination pathway. All these functional genes affect the PRBC's viability during prolonged storage.

DNA replication-related target genes identified in this study are ATAD2 and KATNAL1. ATAD2 coexpresses with genes involved in DNA replication. It is mainly expressed in S-phase cells, localizing to nascent chromatin (replication sites). Similar to ATAD2-depletion, it led to decreased DNA replication [19]. E2F1 is a crucial cell cycle regulator that affects genes encoding proteins involved in DNA repair and apoptosis and proteins that control cell cycle progression through the G1/S transition [20]. DDX5 is a family of enzymes that control the formation and function of ribonucleoproteins in all aspects of RNA metabolism, from synthesis to degradation. Downregulation of DDX5 causes impaired growth and mitochondrial dysfunction [21]. FOXL2 is a transcription factor that plays a crucial role in ovarian development and maintenance. FOXL2 mutations have been implicated in various ovarian disorders, including premature ovarian failure and granulosa cell tumors. Recent studies have also shown that FOXL2 plays a role in cell growth and proliferation. FOXL2 regulates the expression of cyclin-dependent kinase inhibitor 1B (CDKN1B), a key regulator of cell cycle progression. FOXL2 overexpression inhibited cell proliferation in vitro, while FOXL2 knockdown increased cell proliferation. These findings suggest that FOXL2 regulates granulosa cell proliferation by modulating CDKN1B expression [22]. MID1 and RNF6 are involved in the ubiquitination pathway, which is a process that targets proteins for degradation by the proteasome. The ubiquitination pathway regulates many cellular processes, including cell signaling, DNA repair, and protein turnover. Recent studies have suggested that the ubiquitination pathway may also play a role in storing packed red blood cells (PRBCs). MID1 is a ubiquitin E3 ligase involved in various proteins' degradation. MID1 expression increased significantly in PRBCs during storage, and this increase was associated with the degradation of several proteins, including cytoskeletal proteins, membrane-associated proteins, and metabolic enzymes. They also found that MID1 knockdown inhibited the degradation of these proteins and improved the quality of the stored PRBCs [23].

The potential target genes of miRNAs in PRBC storage lesions were involved in structural, biochemical, and immunological (soluble bioactive substances) (Table 3) [13, 14, 24]. Structural was changed in loose membrane phospholipids, shape change, and clumping. A complication of structural changes was cellular necrosis. Biochemicals were changed in the reduction of adenosine triphosphate (ATP), 2,3-diphosphoglycerate (2,3-DPG), nitric oxide (NO), vasodilation, and O_2_ delivery. Immunology was changed in microparticles, cell-free DNA, and bioactive lipids. Complications of immunological changes were inflammation, infection, hypercoagulability, and endothelial injury. The three main changes in PRBC storage resulted in reduced microvascular perfusion and O_2_, accretion in median osmotic fragility (MOF), and death.

According to the U.S. Food and Drug Administration, packed red blood cells (PRBCs) are preserved for 42 days. On average, the units were found to be 21 days old at the time of transfusion [25]. However, other studies found no difference in mortality between the patients who received fresher and older PRBCs [26–29], which stored PRBCs in slow, dangerous changes over time during storage. Although more attention is paid to the damage to PRBC storage, further studies are needed to increase knowledge of modifying PRBC storage in blood banks to assess their potential impact [11].

The downstream regulation mechanism of miR-20a-5p on cell differentiation, proliferation, and apoptosis was clarified. Candidate target genes based on structural, biochemical, and immunological changes need to be further determined and provide a new approach to PRBC storage.

## 5. Conclusions

Increased expression of miR-20a-5p was associated with cell survival, which may affect the downstream regulation and decrease PRBC viability in prolonged storage. Thus, dysregulated miRNA may represent a tremendous potential biomarker to identify blood storage lesions and assess the quality of stored PRBCs.

## Figures and Tables

**Table 1:** Characteristics of blood donors by gender, age, and blood type.

Sex (*n*, %)		Median age (years)	Blood group (*n*, %)			
Male	Female		A	B	AB	O
17 (85)	3 (15)	37 (22-57)	5 (25)	2 (10)	3 (15)	10 (50)

**Table 2:** Twenty-two target genes of miR-20a-5p in PRBC storage lesion.

Gene	Chromosomal location	Gene groups	Functions
*ATAD2*	8q24.13	AAA ATPases	DNA replication, protein degradation, membrane fusion, microtubule severing, peroxisome biogenesis, signal transduction, and regulation of gene expression
*DDX5*	17q23.3	DEAD-box helicases	RNA metabolism
*E2F1*	20q11.22	E2F transcription factors	Cell cycle regulation and synthesis of DNA in mammalian cells
*EPHA5*	4q13.1-q13.2	EPH receptors	Long-term potentiation, angiogenesis, and stem cell differentiation and cancer
*EPHA7*	6q16.1	EPH receptors	Long-term potentiation, angiogenesis, and stem cell differentiation and cancer
*FEM1B*	15q23	Ankyrin repeat domain-containing	Protein-protein interaction domains
*FNBP1L*	1p22.1	F-BAR domain containing	Dimerization and membrane phospholipid binding
*FOXL2*	3q22.3	Forkhead boxes	Cell growth, proliferation, differentiation, and longevity
*HIF1A*	14q23.2	Basic helix-loop-helix proteins	DNA binding
*KATNAL1*	13q12.3	AAA ATPases	DNA replication, protein degradation, membrane fusion, microtubule severing, peroxisome biogenesis, signal transduction, and regulation of gene expression
*KPNA3*	13q14.2	Armadillo repeat-containing	Linking cadherin cell adhesion proteins to the cytoskeleton and transducing WNT signals during embryonic development
*MCC*	5q22.2	EF-hand domain containing	Binds calcium ions
*MID1*	Xp22	Ring finger proteins	Ubiquitination pathway
*MXI1*	10q25.2	Basic helix-loop-helix proteins	DNA binding
*NBEA*	13q13.3	A-kinase anchoring proteins	Binding to the regulatory subunit of protein kinase A (PKA) and confining the holoenzyme to discrete locations within the cell
*NPAS3*	14q13.1	Basic helix-loop-helix proteins	DNA binding
*PTPN21*	14q31.3	FERM domain containing	Plasma membrane and the cytoskeleton
*RNF6*	13q12.13	Ring finger proteins	Ubiquitination pathway
*SAR1B*	5q31.1	ARF GTPase family	Vesicle biogenesis in intracellular traffic
*SCN2B*	11q23.3	V-set domain containing	Myelin membrane adhesion molecules, junction adhesion molecules (JAM), tyrosine-protein kinase receptors, and programmed cell death protein 1 (PD1)
*TANC1*	2q24.2	Ankyrin repeat domain-containing	Protein-protein interaction domains
*TIAM1*	21q22.11	PDZ domain-containing	Anchoring of cell surface receptors (such as CFTR and FZD7) to the actin cytoskeleton via mediators like NHERF and ezrin
		Pleckstrin homology domain containing	Recruiting proteins to different membranes and cellular compartments or enabling them to interact with other components of the signal transduction pathways

**Table 3:** Packed red blood cell (PRBCs) storage lesion.

RBC storage lesion	Structural (i) Lose membrane phospholipids (ii) Shape change (iii) Clumping	Biochemical (i) ↓ ATP (ii) ↓ 2,3-DPG (iii) ↓ NO.	Immunological (soluble bioactive substances) (i) Microparticles (ii) Cell-free DNA (iii) Bioactive lipids

Complications	Cellular necrosis	(i) Impaired vasodilation (ii) O_2_ delivery	(i) Inflammation (ii) Infection (iii) Hypercoagulability (iv) Endothelial injury

Outcomes	(i) ↓ Microvascular perfusion and O_2_ (ii) ↑ MOF and death		

## Data Availability

The preprint data used to support the findings of this study have been deposited in the rejected article of Non-Coding RNA research repository (https://papers.ssrn.com/sol3/papers.cfm?abstract_id=4230900).

## References

[1] Connell N. T. (2016). Transfusion medicine. *Primary Care*.

[2] Carson J. L., Guyatt G., Heddle N. M. (2016). Clinical practice guidelines from the AABB: red blood cell transfusion thresholds and storage. *Journal of the American Medical Association*.

[3] National Institue for Heath and Care Excellence *Blood transfusion*.

[4] Association for the Advancement of Blood & Biotherapies, the American Red Cross, America’s BloodCenters, and the Armed Services Blood Program *Circular of information for the use of human blood and blood components*.

[5] Kalhan T. G., Bateman D. A., Bowker R. M., Hod E. A., Kashyap S. (2017). Effect of red blood cell storage time on markers of hemolysis and inflammation in transfused very low birth weight infants. *Pediatric Research*.

[6] Ng M. S., David M., Middelburg R. A. (2018). Transfusion of packed red blood cells at the end of shelf life is associated with increased risk of mortality–a pooled patient data analysis of 16 observational trials. *Haematologica*.

[7] Flegel W. A., Natanson C., Klein H. G. (2014). Does prolonged storage of red blood cells cause harm?. *British Journal of Haematology*.

[8] Condrat C. E., Thompson D. C., Barbu M. G. (2020). miRNAs as biomarkers in disease: latest findings regarding their role in diagnosis and prognosis. *Cells*.

[9] Sun L., Yu Y., Niu B., Wang D. (2020). Red blood cells as potential repositories of microRNAs in the circulatory system. *Frontiers in Genetics*.

[10] Haberberger A., Kirchner B., Riedmaier I. (2018). Changes in the microRNA expression profile during blood storage. *BMJ Open Sport & Exercise Medicine*.

[11] Chen X., Xie X., Xing Y., Yang X., Yuan Z., Wei Y. (2018). MicroRNA dysregulation associated with red blood cell storage. *Transfusion Medicine and Hemotherapy*.

[12] Yurie H., Ikeguchi R., Aoyama T. (2017). The efficacy of a scaffold-free Bio 3D conduit developed from human fibroblasts on peripheral nerve regeneration in a rat sciatic nerve model. *PLoS One*.

[13] Kim-Shapiro D. B., Lee J., Gladwin M. T. (2011). Storage lesion: role of red blood cell breakdown. *Transfusion*.

[14] Reilly M., Bruno C. D., Prudencio T. M. (2020). Potential consequences of the red blood cell storage lesion on cardiac electrophysiology. *Journal of the American Heart Association*.

[15] Zhu D., Pan C., Li L. (2013). MicroRNA-17/20a/106a modulate macrophage inflammatory responses through targeting signal-regulatory protein *α*. *The Journal of Allergy and Clinical Immunology*.

[16] Huang W., Wu X., Xiang S. (2022). Regulatory mechanism of miR-20a-5p expression in cancer. *Cell Death Discovery*.

[17] Wu H., Pang P., Liu M. D. (2018). Upregulated miR-20a-5p expression promotes proliferation and invasion of head and neck squamous cell carcinoma cells by targeting of TNFRSF21. *Oncology Reports*.

[18] Yang H., Chen Z., Liu Z. (2021). MiR-20a-5p negatively regulates NR4A3 to promote metastasis in bladder cancer. *Journal of Oncology*.

[19] Koo S. J., Fernández-Montalván A. E., Badock V. (2016). ATAD2 is an epigenetic reader of newly synthesized histone marks during DNA replication. *Oncotarget*.

[20] Sheldon L. A. (2017). Inhibition of E2F1 activity and cell cycle progression by arsenic via retinoblastoma protein. *Cell Cycle*.

[21] Xing Z., Russon M. P., Utturkar S. M., Tran E. J. (2020). The RNA helicase DDX5 supports mitochondrial function in small cell lung cancer. *Journal of Biological Chemistry*.

[22] Liu X. L., Meng Y. H., Wang J. L., Yang B. B., Zhang F., Tang S. J. (2014). FOXL2 suppresses proliferation, invasion and promotes apoptosis of cervical cancer cells. *International Journal of Clinical and Experimental Pathology*.

[23] Tzounakas V. L., Dzieciatkowska M., Anastasiadi A. T. (2022). Red cell proteasome modulation by storage, redox metabolism and transfusion. *Blood Transfusion*.

[24] Heddle N. M., Cook R. J., Arnold D. M. (2016). Effect of short-term vs. long-term blood storage on mortality after transfusion. *NEJM*.

[25] Whitaker B., Rajbhandary S., Kleinman S., Harris A., Kamani N. (2016). Trends in United States blood collection and transfusion: results from the 2013 AABB blood collection, utilization, and patient blood management survey. *Transfusion*.

[26] Alexander P. E., Barty R., Fei Y. (2016). Transfusion of fresher vs older red blood cells in hospitalized patients: a systematic review and meta-analysis. *Blood*.

[27] Chai-Adisaksopha C., Alexander P. E., Guyatt G. (2017). Mortality outcomes in patients transfused with fresher versus older red blood cells: a meta-analysis. *Vox Sanguinis*.

[28] Cooper D. J., McQuilten Z. K., Nichol A. (2017). Age of red cells for transfusion and outcomes in critically ill adults. *NEJM*.

[29] Rygård S. L., Jonsson A. B., Madsen M. B. (2018). Effects of shorter versus longer storage time of transfused red blood cells in adult ICU patients: a systematic review with meta-analysis and trial sequential analysis. *Intensive Care Medicine*.

